# Isolated tuberculous orchitis: A mimicker of testicular malignancy

**DOI:** 10.4103/0970-1591.65404

**Published:** 2010

**Authors:** Joseph Paul, Sriram Krishnamoorthy, Marie Teresa, Santosh Kumar

**Affiliations:** Department of Urology, Christian Medical College, Vellore, India; 1 Department of Pathology, Christian Medical College, Vellore, India

**Keywords:** Filariasis, testicular tumor, tuberculous orchitis

## Abstract

Isolated tuberculous orchitis is a rare entity. The coexistence of tuberculosis of the testis with filarial worm infestation is even more rare. In this report, we present a case of tuberculous involvement of the testis, associated with filarial worm infestation in the spermatic cord that presented as a testicular tumor. A 55-year-old male presented with unilateral testicular swelling of four months duration. As the clinical evaluation was suggestive of testicular malignancy, he underwent a right high orchidectomy. The histopathology report revealed isolated tuberculous orchitis without epididymal involvement along with filarial infestation of the spermatic cord.

## INTRODUCTION

Genitourinary tuberculosis is the second most common form of extra pulmonary tuberculosis.[1] Genital tuberculosis other than tuberculous (TB) epididymitis is rare. Isolated TB orchitis without epididymal involvement is even more rare.[2] It is also rare to find a case of TB testis with coexistent filarial infection of the spermatic cord. To the best of our knowledge, this is the first case reported in literature where tuberculosis is seen to coexist with filarial infestation.

## CASE REPORT

**Figure 1:**
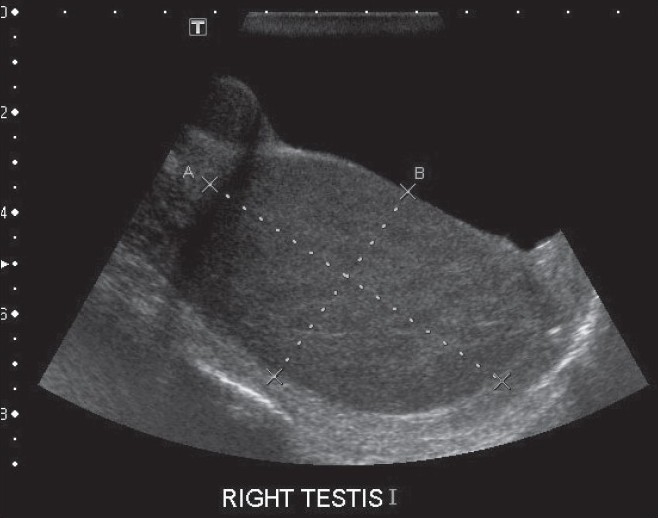
Ultrasound of the right testis showing homogenously enlarged testicular parenchyma

**Figure 2:**
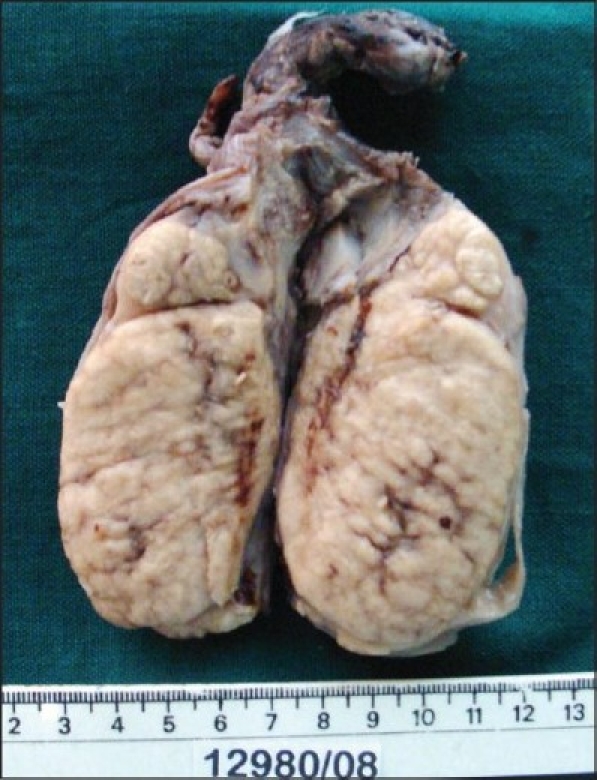
Cut section of the gross specimen showing the testicular parenchyma replaced with granuloma

A 55-year-old male presented with a painless swelling of the right testis of four months duration. He had recurrent low-grade fever for the past 1 month, not associated with chills or rigor. He did not have any loin pain or bothersome voiding difficulties. He was not treated for TB in the past nor did he have a family history of TB. There was no trauma to the scrotum. The general examination was unremarkable. Upon examination, there was an 8 × 4 cms hard and nontender swelling in the right testis with an irregular surface. The left testis, both epididymes and the cord structures were clinically normal. The ESR was 45 mm in one hour, total white blood cell (WBC) count was 3400 per cub mm with a differential count of 72 polymorphs and 25 lymphocytes. Urine microscopy revealed 10-12 red blood cells(RBC)/high power field and urine culture was sterile. Serum LDH was 947 U/L but beta HCG (<1 mIU/mL) and AFP (1.71 IU/mL) were normal. The chest X-ray was normal. An ultrasonogram of the scrotum revealed a 7 × 4.5 cms hypoechoic lesion in the right testis [Figure 1]. The left testis and both epididymes were normal. An ultrasound examination of the abdomen was normal. A clinical diagnosis of testicular tumor was made and the patient underwent a right inguinal orchidectomy. Cut section of the gross specimen showed testicular parenchyma replaced by granuloma [Figure 2].

**Figure 3:**
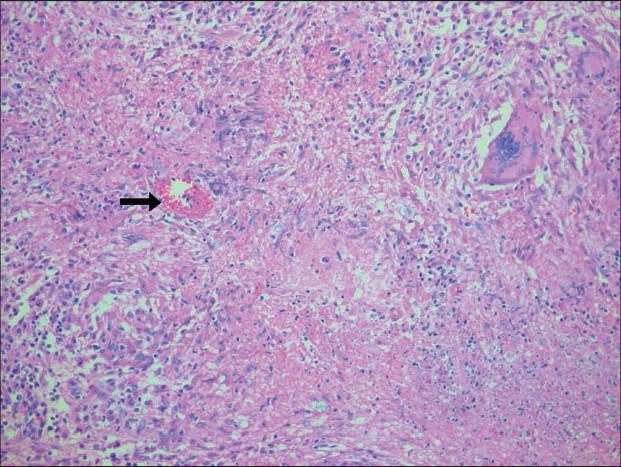
Microscopic picture showing testicular parenchyma replaced by caseating granuloma (H and E, ×100)

**Figure 4:**
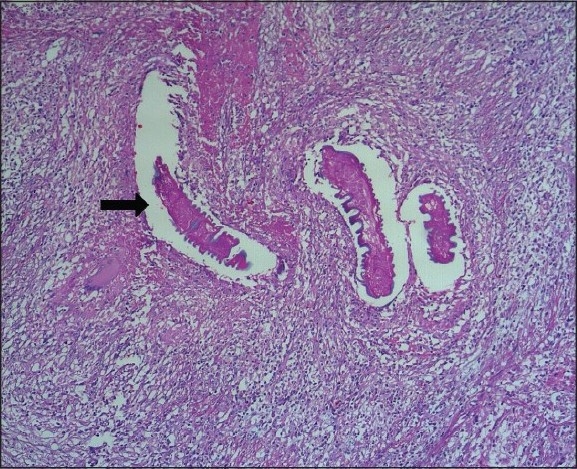
Microscopic picture showing an adult fi larial worm in the lymphatic channel (H and E, ×100)

Histopathological examination revealed a caseating granulomatous lesion, consistent with TB orchitis. Testicular parenchyma was replaced by caseating granulomas composed of epithelioid histiocytes, lymphocytes, and Langhans' multinucleate giant cells and surrounded by lymphocytes and plasma cells [Figure 3]. There was interstitial fibrosis. The granulomatous infiltrate extended onto the tunica albuginea. Occasional atrophic seminiferous tubules were also present. There was also an adult filarial parasite in a dilated lymphatic channel of the right spermatic cord [Figure 4].

## DISCUSSION

Genitourinary TB is the second most common site of involvement among extra-pulmonary TB.[1] About 28% of these patients will have isolated genital involvement.[4] The usual site of involvement is the epididymis, which occurs haematogenously or by a retrocanalicular haematogenous pathway from an infected prostate. If the infection goes unchecked, it can involve the testis. In a series of 96 cases of genital TB, Edward could not find any primary testicular TB; all had the primary involvement of the epididymis.[5] The first sign of tuberculous epididymitis is the appearance of a hard nodule at the cauda of the epididymis. This nodular induration later involves the entire epididymis, then the vas for a varying distance, and lastly the testis.[6] But, isolated involvement of testis is very rare. The possible etiology of isolated tuberculous orchitis is that rarely the infection of the testis could be by hematogenous route rather than the usual direct extension from the epididymis. In one reported case of isolated TB orchitis, the patient presented with scrotal ulceration.[3]

In our case, the presentation was typically mimicking a testicular tumor. The chest X-ray was normal. He did not have any constitutional symptoms of TB. The urine smear was negative for acid fast bacilli. All these features confirmed the diagnosis of isolated TB orchitis. Genital filariasis commonly presents as a secondary vaginal hydrocele with an associated epididymo-orhitis. Savio *et al*, have reported filarial involvement of the tunica of testis.[7] Kundu *et al*, have reported a case of testicular TB diagnosed by fine needle aspiration cytology. The lesion regressed with antituberculous treatment.[8] Kumar *et al*, have reported a case of acquired immunodeficiency syndrome (AIDS) presenting as testicular TB. ^9^ This is the first case report in literature of an isolated TB of the testis with coexistent filarial infestation of the spermatic cord. We also want to convey the message that in the atypical age group of patients presenting with testicular swelling from endemic areas of tuberculosis and filariasis, an infective etiology should be considered.
